# The quality-of-care agenda in fragile, conflict-affected and vulnerable settings

**DOI:** 10.2471/BLT.21.285627

**Published:** 2021-03-01

**Authors:** Matthew Neilson, Sheila Leatherman, Shamsuzzoha Syed

**Affiliations:** a Department of Integrated Health Services, World Health Organization, Avenue Appia 20, 1211 Geneva 27, Switzerland.; b Gillings School of Global Public Health, University of North Carolina, Chapel Hill, United States of America.

Inadequate quality of care claims between 5.7 million and 8.4 million lives each year in low- and middle-income countries.^1^ Access to quality care could prevent 2.5 million deaths from cardiovascular disease, 1 million newborn deaths, and half of all maternal deaths annually; yet many settings lack even basic infrastructure such as water in health facilities.^2^ In 2018, three global reports affirmed the need for improving quality of care as countries pursue universal health coverage.^1^^–^^3^ While a significant body of knowledge exists on how to address quality, action is still required to accelerate global progress,^4^ particularly in fragile, conflict-affected and vulnerable settings, home to almost a quarter of the world’s population.^5^ Studies estimate that such settings account for 60% of preventable maternal deaths, 53% of deaths in children younger than 5 years, and 45% of neonatal deaths.^6^ Furthermore, the coronavirus disease 2019 pandemic has highlighted the challenges the most vulnerable face in realizing their right to quality health care.

In response to this need for action, the World Health Organization (WHO) has worked with partners to develop a practical approach to address quality in fragile, conflict-affected and vulnerable settings.^7^ These efforts have culminated in a technical package comprising an action-focused guide^8^ and tools compendium.^9^

The technical package builds on WHO’s established approach to national quality policy and strategy.^10^^,^^11^ The package is designed for the complexities of delivering health services in such settings. Over a 2-year period, Gillings School of Global Public Health at the University of North Carolina, United States of America, in collaboration with WHO, undertook foundational research to understand the evidence base and implementation experience around what works to improve quality in these settings.^12^ This research was supplemented by several rounds of consultation with experts to ground the technical package in a pragmatic understanding of the factors that determine successful implementation.

*Quality of care in fragile, conflict-affected and vulnerable settings: taking action* presents a flexible, non-prescriptive approach to support action planning for quality in fragile, conflict-affected and vulnerable settings.^8^ Implementation will be different across diverse settings, also depending on the scope of executing organizations. Action may be taken by: individual providers or humanitarian partners for services that they provide; multiple stakeholders pursuing a coordinated quality agenda; national governments, in settings where they have adequate control and capacity; and subnational governments addressing a specific geographical area, complementing the broader national quality strategy where it exists.

The technical package outlines eight elements for quality action planning: service priorities and quality goals; shared local understanding of quality; stakeholder mapping and engagement; situational analysis; governance for quality; interventions for quality improvement; health information systems and quality assessment; and quality measurement. 

Key actions required for each of these eight elements are described in the technical package. In practice, the eight elements are interdependent. In most settings, they can be addressed together in a process involving initial engagement efforts and identification of a team to lead the process; assessment of needs, challenges and assets; building of consensus and planning of activities; and steps to validate the plan and secure leadership support for implementation. Action should not wait until a plan is finalized; rather, starting early implementation is important to tackle urgent quality issues, demonstrate success of improvement interventions and generate learning to inform development and roll-out of the action plan. Local expertise and experience are critical in adapting the approach to local settings.

One of the eight elements – interventions for quality improvement – is particularly important. This section presents a series of evidence-based interventions organized under five areas: ensure access and basic infrastructure for quality; shape the system environment; reduce harm; improve clinical care; and engage patients, families and communities. The tools and resources compendium is based on this interventions list, providing a curated set of implementation tools gathered during an extensive scoping exercise.^9^

This WHO technical package provides a starting point for multi-actor efforts to address quality of care in fragile, conflict-affected and vulnerable settings. We call on stakeholders across the health, humanitarian and development sectors to move forward on three fronts. First, implement this technical package across such settings, prioritizing early implementation of quality interventions while building for longer-term transformation. Second, support consideration of quality in these settings within routine health systems planning, humanitarian response and development assistance. Third, conduct practical operational research to identify what interventions most reliably and efficiently deliver benefits within inevitable resource constraints.
